# Real-time predictors and consequences of binge eating among adults with type 1 diabetes

**DOI:** 10.1186/s40337-019-0237-3

**Published:** 2019-03-18

**Authors:** Ashley A. Moskovich, Natalia O. Dmitrieva, Michael A. Babyak, Patrick J. Smith, Lisa K. Honeycutt, Jan Mooney, Rhonda M. Merwin

**Affiliations:** 1 Duke University Medical Center, DUMC Box 3842, Durham, NC 27712 USA; 2 Northern Arizona University, Flagstaff, AZ USA

**Keywords:** Type 1 diabetes, Eating disorders, Binge eating, Ecological momentary assessment, EMA

## Abstract

**Background:**

Objective binge eating (OBE) is common among individuals with type 1 diabetes (T1D) and may have negative consequences for glycemic control. Recent studies have suggested that diabetes distress (i.e., emotional distress specific to diabetes and living with the burden of management) is a distinct emotional experience among individuals with diabetes. Preliminary studies have found diabetes distress is associated with eating disorder symptoms and poor glycemic control. The aim of the current study was to examine real-time emotional precursors and consequences of OBE in adults with T1D (i.e., general negative affect, specific emotional states and diabetes distress) using ecological momentary assessment methods. We also explore the impact of OBE on 2-h postprandial glycemic control relative to non-OBE eating episodes.

**Methods:**

Adults with T1D (*N* = 83) completed 3-days of ecological momentary assessment assessing mood and eating behavior using a telephone-based survey system. Participants were prompted to rate momentary affect, including level diabetes distress, at random intervals and reported on eating episodes. Participants also wore continuous glucose monitors allowing for ongoing assessment of glycemic control. Multi-level modeling was used to examine between- and within-person effects of momentary increases in emotions prior to eating on the likelihood of OBE and the impact of OBE on postprandial blood glucose. Generalized linear mixed models examined whether change in post-meal affect differed between OBE and non-OBE episodes.

**Results:**

Participants were predominately middle-aged (Mean = 42; SD = 12.43) Caucasian (87%) females (88%) reporting clinically significant eating disorder symptoms (76%). Nearly half of the sample (43%) reported OBE during the 3-day study period. The between-person effect for negative affect was significant (OR = 1.93, *p* < .05), indicating a 93% increased risk of OBE among individuals with higher negative affect compared to individuals with average negative affect. Between-person effects were also significant for guilt, frustration and diabetes distress (OR = 1.48–1.77, *p*s < .05). Analyses indicated that mean change in post-meal negative affect was significantly greater for OBE relative to non-OBE episodes (*B* = 0.44, *p* < .001). Blood glucose at 120 min postprandial was also higher for OBE than for non-OBE episodes (*p* = .03).

**Conclusions:**

Findings indicate that individuals who tend to experience negative affect and diabetes distress before eating are at increased risk of OBE at the upcoming meal. Results also suggest that engaging in binge eating may result in greater subsequent negative affect, including diabetes distress, and lead to elevated postprandial blood glucose levels. These findings add to a growing literature suggesting diabetes distress is related to eating disordered behaviors among individuals with T1D.

## Plain english summary

Binge eating is common among individuals with type 1 diabetes (T1D) and may negatively impact glycemic control. Existing eating disorder treatments are less effective for individuals with T1D, suggesting the need to better understand factors that may contribute to binge eating in this population. Previous research indicates that people are more likely to binge eat when they are feeling negative emotions. However, whether there are different emotional antecedents or consequences for T1D patients is not known. The current study tested whether increases in negative affect, including diabetes distress (i.e., emotional distress specific to living with diabetes), increased the likelihood of binge eating among adults with T1D. Eighty-three adults with T1D provided information about their emotions and eating behavior throughout the day for 3 days using a telephone-based survey system. Findings indicated individuals who reported higher levels of guilt, frustration and diabetes distress before eating episodes were more likely to binge eat than individuals who reported average levels of these emotions. Results also indicated that people felt worse (generally and about diabetes) and had higher blood glucose levels after binge eating. Findings suggest that interventions focused on helping individuals cope with negative emotions and diabetes distress may be helpful to incorporate into treatments for binge eating.

## Background

Eating problems are common among individuals with type 1 diabetes [1–3]. This includes binge eating, dietary restriction and compensatory behaviors observed in the general population (e.g., self-induced vomiting) along with the unique capacity to restrict insulin for weight control. Studies show that disordered behaviors are associated with poor metabolic control and diabetes complications even when full diagnostic criteria for an eating disorder is not met (e.g., binge eating disorder, bulimia nervosa) [1, 3–5].

Up to 45–80% of young women with type 1 diabetes report binge eating behavior [6, 7], and many engage in this behavior frequently [3]. For example, a recent study found that 56% of adolescents completing a national survey reported binge eating at least once during the past 14 days [3]. Objective binge eating (OBE), defined as a loss of control over eating while consuming an objectively large amount of food [8], makes it challenging to manage diabetes. Most notably, individuals may struggle to accurately count carbohydrate and estimate bolus insulin needs when eating is uncontrolled. Binge eating may also motivate the use of dangerous behaviors to compensate for excessive calories consumed, further disrupting glycemic control (e.g., intentionally restricting insulin to induce glycosuria; the excretion of glucose into the urine) [9]. Poor glycemic control is associated with an array of diabetes-related medical complications (e.g., neuropathy, retinopathy) [9, 10], highlighting the importance of addressing OBE in this patient population.

Despite the high prevalence and clinical significance of OBE in type 1 diabetes, it remains an understudied problem and effective treatments are lacking. Data suggests that conventional outpatient eating disorder treatments (developed for nondiabetic individuals) are less effective for individuals with type 1 diabetes [11–14]. One treatment study found that individuals continued to binge eat and restrict insulin, even when there were improvements in weight and shape concerns [14]. This indicates the need to better understand the factors associated with OBE in this unique patient population in order to develop more effective interventions.

A large body of empirical work in the general population suggests that negative affect is associated with OBE among individuals without diabetes [15, 16]. Previous studies have demonstrated that individuals who engage in OBE tend to report higher levels of depression and anxiety in general [16–18], and experience momentary increases in negative affect prior to binge eating episodes. When examining specific emotional states, guilt, sadness, and anger tend to be the strongest predictors of binge eating [19–22]. Studies examining affect post binge eating have mixed results. While some studies report that negative affect increases after binge eating [15, 21], others suggest that binge eating may actually temporarily decrease negative affect thereby functioning as a maladaptive emotion regulation strategy [19, 20, 22, 23].

At least one study has found individuals with type 1 diabetes with OBE report greater anxiety and depression than type 1 diabetes patients who do not engage in OBE [24]. However, there are no studies that have tested momentary changes in affect that may precede or follow an episode of binge eating in this high-risk patient population. Thus, whether emotional precursors or consequences of OBE are the same for individuals with type 1 diabetes as in the general population is unknown. This may include differences in general emotional states or more uniquely, diabetes distress (i.e., emotional distress specific to diabetes and living with the burden of management), which has been identified as a distinct emotional state experienced by individuals with diabetes [25–27].

For individuals with diabetes, maintaining control over eating is not only about controlling weight but also about preventing immediately life-threatening circumstances (e.g., diabetic ketoacidosis) and staving off future long-term diabetes-related complications. Thus, mealtimes may be particularly emotionally evocative, evoking negative affect generally, and specifically feelings of fear, anger and guilt associated with diabetes and living with the burden of diabetes management. Diabetes distress is increasingly recognized as a factor associated with poor management in type 1 diabetes [27, 28]. A few studies suggest diabetes distress is associated with eating disorder symptoms and taking less insulin than is needed [26, 29–31]. However, whether diabetes distress is functionally-related to OBE, increasing risk for this behavior in real-time, a consequence of OBE, or neither, has not been determined.

The current study examined real-time precursors and consequences of OBE in adults with type 1 diabetes using ecological momentary assessment methods. We examined general negative affect as a predictor of OBE, as well as specific emotional states and diabetes distress. We also established both the between- and within-person effects in each predictor (i.e., distinguished between individual differences in negative affect from potential additional impact of a momentary increase in negative affect) on OBE episode likelihood. Finally, we examined the impact of binge eating on glycemic control by examining blood glucose 2 h postprandial for OBE episodes versus non-OBE episodes. These findings may shed light on behavior patterns and treatment targets for this high-risk patient population.

## Methods

### Participants

Participants were recruited from two medical centers in the Southeast United States and the surrounding area as part of a larger study investigating eating disorder symptomatology among individuals with type 1 diabetes (see [31, 32]). Participants were adults between the ages of 18 and 65 years diagnosed with type 1 diabetes without hypoglycemic unawareness (as assessed via the Gold method [33]) or cognitive disabilities that interfered with their capacity to manage diabetes independently. Individuals with clinically significant eating disorder symptomatology (as indicated by a score of ≥20 on the Diabetes Eating Problems Survey-Revised (DEPS-R) [34] described in more detail below) were recruited first to answer primary research questions of the larger investigation (see Merwin et al., 2018 [32]). After the initial recruitment target was surpassed, enrollment was opened to individuals with DEPS-R scores below 20 to capture the full range of eating disorder symptomatology (see Merwin et al., 2015 [31]). The final sample consisted of 83 individuals, including 63 with DEPS-R ≥ 20.

### Procedure

As described in previous papers (see [31, 32]), participants completed 3 days of ecological momentary assessment of mood and eating behavior using a telephone-based survey system. Interstitial glucose levels were monitored throughout the assessment period using blinded continuous glucose monitoring (CGM).

Eligible participants presented to the laboratory on two separate days. On Day 1, participants completed self-report measures of their illness history and had a blood draw to determine hemoglobin A_1c_ (HbA_1c_). They had a glucose sensor placed and were then trained on the momentary assessment procedures which included completing surveys at random intervals throughout the day and initiating survey entries for meals/snacks (described in detail below). Participants returned to the laboratory 3 days later to have their glucose sensor removed and data downloaded using specialized software. They also completed additional self-report measures not relevant to the current study. Procedures were approved by the Duke University Health System Institutional Review Board (IRB) and all participants documented informed consent before participation in the study protocol.

### Assessments

#### Diabetes eating problems survey-revised (DEPS-R; [34])

The DEPS-R is a 16-item self-report assessment of problematic eating attitudes and behaviors specifically tailored to individuals with diabetes. Items measure how often the individual has experienced each attitude and behavior over the past 4 weeks using a 6-point scale ranging from “Never” to “Always”. Sample items include “I feel that my eating is out of control”, “I would rather be thin than have good control of my diabetes” and “After I overeat, I skip my next insulin dose.” DEPS-R scores range between 0 and 80 and scores ≥20 have been associated with higher HbA_1c_ [34]). The DEPS-R has demonstrated excellent internal consistency (Chronbach’s α = 0.86–0.89), good construct validity as evidenced by associations with diabetes distress and eating disorder symptoms, and external validity [34, 35].

#### Ecological momentary assessment (3 days)

Participants received randomly generated phone calls from IfByPhone®, an automated telephone system, at the rate of 1–2 times an hour between the hours of 8 am and 10 pm. Participants also placed calls to the survey system to report meals/snacks and were asked to do so immediately after eating. At each call, participants completed brief surveys (taking less than 1–2 min to complete) on their current emotion or mood and eating and type 1 diabetes management behavior.

At each call, participants were asked to provide momentary ratings of their affective state (*happy, sad, frustrated, angry, anxious or nervous, guilty or disgusted with yourself*) using a scale from 1 to 6 (e.g., “On a scale from 1-6, how *sad* do you feel?”). Current level of diabetes distress was also assessed by the following question: “How upset do you feel about your diabetes or diabetes management?” (1 = Not at All, 6 = Very Much). For calls reporting eating, participants were also asked to indicate the time they started eating and answer questions about their eating behavior. Relevant to the current study were questions assessing whether or not OBE was present including: “Did you eat a *large amount of food*, more than would be typical of others in a similar situation?” and “Did you experience a *loss of control* over your eating?” . For the first item (Large amount of food), participants responded with key presses indicating 1 = Yes, I ate a large amount of food or 2 = No). For the second item (Loss of control), participants responded using a 6-point Likert scale (1 = Not at all, indicating no loss of control; 6 = Very much). Later, this item was changed to dichotomous for ease of administration and analysis (1 = Yes, loss of control present, 2 = No loss of control). OBE was determined to be present when participants indicated that “Yes” they had eaten a large amount of food for the situation *and* “Yes” they had experienced a loss of control over eating (i.e., reporting anything other than 1 = No loss of control for the scaled item; or “Yes” to the dichotomous item).

Participants received specific training on the study definitions of a “large amount of food” (i.e., an amount of food that is definitely larger than what most individuals would eat in a similar situation) and “loss of control over eating” (i.e., a feeling that one cannot stop or control eating) as defined by the Diagnostic and Statistical Manual of Mental Disorders – 5 [8]. The study coordinator reviewed the definitions with all individuals and provided examples. Participants received a training handbook that included the definitions and examples to refer to as needed throughout the 3-day assessment period.

#### Continuous glucose monitoring (CGM)

The Medtronic CGMS® iPro™ or iPro2™ was used for CGM. Trained study staff inserted Medtronic glucose sensors under participants’ abdominal skin and then connected the sensor to small, lightweight monitors. Monitors lay flat on the skin of abdomen, attached by a small adhesive patch. Participants were able to engage in all normal activities after sensor placement, including swimming and bathing. Sensors sampled interstitial glucose levels continuously and transferred 5-min averages to the monitors for storage. Participants were blinded to their glucose values, but did continue to check their blood glucose with finger sticks a minimum of 3 times a day (as needed for CGM calibration and ongoing management of their diabetes) using a One Touch meter and strips that we provided. The mean absolute difference percentage of 9.9 indicated that CGM calibration accuracy was good. CGM data was downloaded by specialized software after completion of the 3-day assessment period.

### Data analytic strategy

Eating reports, random prompts and CGM data were time synced for analyses.

#### Level of participation

Level of participation during the 3-day assessment period was determined by calculating the percentage of random calls completed and total number of eating episodes reported.

#### Emotional predictors of binge eating

We used multi-level modeling to examine the effects of momentary increases in emotions prior to eating on the likelihood of OBE episode. This allowed us to examine both within and between-person effects while also accounting for the nesting of observations [36–38]. Analyses were restricted to eating episodes with random prompt affect ratings within 60 min prior to eating. We controlled for the time between the affect report and start of eating. We created a composite negative affect variable by taking the mean responses of *sad, frustrated, angry, anxious or nervous, and guilty or disgusted with self* to first examine the overall effect of negative affect on OBE. All individual emotional states, including happiness and diabetes distress were then examined as independent predictors of OBE.

We used a two-level generalized linear mixed modeling (SAS GLIMMIX) strategy with random intercepts to predict the likelihood of the dichotomous OBE variable. Models were estimated with Maximum Likelihood adaptive Gauss-Hermite quadrature, the logit link function, binary distribution, between-within denominator degrees of freedom.

#### Change in post-meal affect

Generalized linear mixed models were used to examine whether change in post-meal negative affect differed significantly between OBE and non-OBE episodes. Analyses were constrained to eating episodes with random prompt affect ratings within 60 min before and after eating. We controlled for pre-meal levels of affect. We first compared OBE to non-OBE eating episodes on post-meal negative affect using the composite negative affect variable. Follow-up analyses for individual affect states, including diabetes distress, were then conducted.

#### OBE and glycemic control

We estimated a two-level linear mixed-model to examine the effect of OBE on 120-min postprandial blood glucose. Predictor variables included OBE (coded as present or absent) and pre-meal blood glucose, with the participant serving as the clustering variable. Thus, our analysis compared the impact of OBE to the impact of all other eating episodes, including normal eating, overeating, and subjective binge eating (i.e., eating events during which an individual experiences a loss of control over eating, but does not consume an objectively large amount of food [39]).

## Results

### Sample characteristics

Eighty-three adults with type 1 diabetes participated in the current study. The sample was predominately female (88%) and Caucasian (87%) with a mean age of 41.9 (*SD* = 12.43; Range 18–68). See Table 1 for additional demographic information. Data analyses excluded nine individuals who either did not complete the 3-day assessment (*n* = 5) or had unusable data due to technical problems (*n* = 4).

**Table 1:** *Participant Demographics (N = 83)*

Characteristic	*Mean* (*SD*) or %
Age (yrs.)	41.89 (12.43)
Sex (% female)	88.00
Race/Ethnicity (%)	
Caucasian/White	86.7
African-American/Black	10.8
Asian/Pacific-Islander	1.2
Hispanic	1.2
Marital Status (%)	
Never Married	22.9
Married	63.9
Separated/Divorced	12.0
Widowed	1.2
Highest Level of Education (%)	
High school graduate or GED	6.0
Some college/technical school	19.3
Bachelor’s Degree	54.2
Graduate degree	20.5
Age at Type 1 Diabetes Diagnosis (Yrs.)	18.5 (10.7)
Duration of Type 1 Diabetes (Yrs.)	23.4 (13.4)
Treatment Regimen (%)	
Insulin Pump Therapy	62.7
Multiple Daily Injections	37.3
HbA_1c_ (mean (SD))	8.8 (2.3)

### Level of participation

Level of participation was high. Participants on average responded to 96% of the random prompts and reported 4 eating episodes per day. Participants reported eating 1002 eating episodes during the 3-day period, 80 (8%) of which were characterized as OBE episodes. Nearly half of the sample (44%) reported at least one episode of OBE during the 3 days.

### Emotional predictors of binge eating

The current analyses examined the reported eating episodes with random prompt affect ratings within 60 min of eating (*n* = 659). This included 55 OBE episodes reported by participants. As demonstrated in Table 2, there was a between-person effect for negative affect 60 min prior to a meal predicting OBE (*OR* = 1.93, *p* = .02, 95% CI = 1.09, 3.41), indicating a 93% increased risk of OBE among individuals with higher negative affect compared to individuals with average negative affect. The odds ratio indicates that for every 1-point increase in the negative affect score, the odds of OBE nearly doubled.

**Table 2:** Fixed Effect Estimates for Affect Predicting Subsequent Binge Eating Episode

Parameter	*B*	*SE*	*Odds Ratio*	*95% CI*
Intercept	− 3.63***	0.5		
Lag Time	1.02	0.7	2.8	0.7–11.2
Negative Affect (BP)	0.66*	0.3	1.9	1.1–3.4
Negative Affect (WP)	−0.43	0.3	0.7	0.4–1.1
Intercept	−3.60***	0.5		
Lag Time	1.01	0.7	2.7	0.7–10.9
Happy (BP)	−0.13	0.2	0.9	0.6–1.4
Happy (WP)	0.06	0.2	1.1	0.8–1.5
Intercept	−3.65***	0.5		
Lag Time	1.03	0.7	2.8	0.7–11.3
Sad (BP)	0.21	0.4	1.2	0.6–2.6
Sad (WP)	−0.42^†^	0.2	0.7	0.4–1.0
Intercept	−3.58***	0.5		
Lag Time	0.92	0.7	2.5	0.6–10.1
Angry (BP)	0.46	0.2	1.6	0.9–2.9
Angry (WP)	−0.38^†^	0.3	0.7	0.5–1.0
Intercept	−3.62***	0.5		
Lag Time	1.00	0.7	2.7	0.7–10.9
Frustrated (BP)	1.35*	0.4	1.7	1.1–2.6
Frustrated (WP)	−0.23	0.2	0.8	0.6–1.1
Intercept	−3.65***	0.5		
Lag Time	1.07	0.7	2.9	0.7–11.9
Anxious/Nervous (BP)	0.46^†^	0.2	1.6	1.0–2.5
Anxious/Nervous (WP)	−0.20	0.2	0.8	0.6–1.2
Intercept	−3.62***	0.5		
Lag Time	1.02	0.7	2.8	0.7–10.9
Guilty/Disgusted With Yourself (BP)	0.57*	0.2	1.8	1.1–2.8
Guilty/Disgusted With Yourself (WP)	0.07	0.2	1.1	0.8–1.4
Intercept	−3.65***	0.5		
Lag Time	1.05	0.7	2.9	0.7–11.4
Upset about Diabetes (BP)	0.40*	0.2	1.5	1.1–2.1
Upset about Diabetes (WP)	0.15	0.2	1.2	0.8–1.7

*BP* between persons effects, *WP* within-persons effects

^†^*p* < .10. *p < .05. **p < .01. ***p < .001

Analyses testing specific affect states as predictors of OBE indicated significant between-person effects for *guilty or disgusted with yourself* (*OR* = 1.77, *p* = .01, 95% CI = 1.13, 2.77), *frustrated* (*OR* = 1.71 *p* = .01, 95% CI = 1.13, 2.59), and *diabetes distress* (*OR* = 1.48, *p* = .02, 95% CI = 1.07, 2.07).

There was not a significant within-person effect of negative affect on OBE (*OR* = 0.65, *p* = .11, 95% CI = 0.39, 1.10), indicating that a momentary increase does not confer additional risk over and above the effect of individual differences in negative affect. In exploratory analyses, there were non-significant within-person trends for *sad (OR* = 0.66, *p* = .07, 95% CI = 0.42, 1.04) and *angry* (*OR* = 0.69, *p* = .08, 95% CI = 0.45, 1.04). See Table 2.

### Change in post-meal affect

Analyses were conducted with the 520 eating episodes that had affect reports within 60 min before and after eating. As shown in Table 3, results indicated that, after controlling for pre-meal negative affect, the increase in negative affect was greater when binge eating occurred than when it did not, *B* = 0.44, *SE* = 0.08 *p* < .001.

**Table 3:** Fixed Effect Estimates of OBE Episodes Predicting Mean Change in Affect Relative to Non-OBE Episodes

Parameter	*B*	*SE*	*DF*	t
Intercept	1.21***	0.1	75	10.1
Pre Negative Affect	−0.50***	0.1	442	−9.4
Post OBE Δ Negative Affect	0.44***	0.1	442	−5.4
Intercept	1.63***	0.2	74	7.1
Pre Happy	−0.57***	0.1	443	−10.7
Post OBE Δ Happy	−0.13	0.2	443	0.8
Intercept	1.21***	0.1	75	9.4
Pre Sad	−0.59***	0.1	443	−12.9
Post OBE Δ Sad	0.39***	0.1	443	−3.6
Intercept	1.28***	0.1	75	9.4
Pre Angry	−0.71***	0.1	443	−15.3
Post OBE Δ Angry	0.27*	0.1	443	−2.5
Intercept	1.66***	0.2	75	9.2
Pre Frustrated	−0.59***	0.1	442	−11.9
Post OBE Δ Frustrated	0.60***	0.2	442	−3.9
Intercept	1.06***	0.1	75	7.3
Pre Nervous/Anxious	−0.52***	0.1	442	−10.7
Post OBE Δ Nervous/Anxious	0.27*	0.1	442	−2.2
Intercept	1.42***	0.2	75	9.0
Pre Guilty/Disgusted With Yourself	−0.47***	0.1	442	−10.1
Post OBE Δ Guilty/Disgusted With Yourself	0.59***	0.1	442	−4.8
Intercept	1.57***	0.2	75	7.4
Pre Upset About Diabetes	−0.49***	0.1	442	−8.1
Post OBE Δ Upset About Diabetes	0.30*	0.1	442	−2.4

All models control for pre-meal levels of affect. *OBE* objective binge eating

*p < .05. **p < .01. ***p < .001

Analyses examining specific post-meal affective states indicated that changes in affect were significantly greater for OBE episodes relative to non-OBE episodes for all emotions except *happiness*. After controlling for pre-meal level of affect, individuals reported increased levels of *sadness*, *anger*, *frustration*, *anxiety or nervousness*, *guilt or disgust with yourself*, and *diabetes distress* following OBE compared to non-OBE episodes (*Bs*: 0.27–0.60, all *p*s < .05). The strongest effects were for *frustrated* (*B* = 0.60, *SE* = 0.16, *p* < .001) and *guilty or disgusted with yourself* (*B* = 0.59, *SE* = 0.12, *p* < .001). See Table 3.

### OBE and glycemic control

Blood glucose at 120 min postprandial was higher for OBE (*M* = 213 mg/dL, 95% CI = 191, 234), than for non-OBE episodes (*M* = 188 mg/dL, 95% CI = 179, 198), *p* = .03.

## Discussion

The current study examined real-time emotional predictors and consequences of binge eating during 3-days of ecological momentary assessment in adults with type 1 diabetes reporting a range of eating disorder symptomatology. Of the 1002 eating episodes reported by participants, 80 were classified as OBE episodes and 43% of participants engaged in binge eating at least once during the 3-day assessment period. Results indicated increased odds of OBE among individuals with higher levels of pre-meal negative affect and increased emotional distress and 2-h postprandial blood glucose following OBE relative to non–OBE episodes.

Elevated blood glucose levels may be the result of difficulty determining amounts of carbohydrate consumed and approximating insulin response to the carbohydrate load or intentional insulin restriction to compensate for calories consumed. Overall, results highlight the importance of helping individuals with type 1 diabetes develop skills to cope with emotional distress in order to manage diabetes and achieve optimal glycemic control.

In the current study, individuals with type 1 diabetes who reported higher average levels of frustration, guilt and diabetes distress within 60 min of eating were more likely to engage in OBE than their peers who scored lower on negative affect. Individuals with elevated levels of negative affect before eating may be struggling to cope with managing a chronic illness that impacts every meal. For example, individuals may feel frustrated about their pre-meal blood glucose and their ability to control their blood glucose and eating well enough to achieve optimal glycemic targets. They may also feel as though they have to impose strict dietary rules to control blood glucose and may feel frustrated that diabetes prevents them from eating what they would like or feel guilt and distress about what they are planning to eat. These individuals may turn toward binge eating as a way to cope with these difficult feelings, or alternatively, give up and abandon efforts at managing diabetes altogether by eating unrestricted amounts of food. Evidence suggests that this leads to more, not less distress (including specifically about diabetes), which may in part be due to the negative impact losing control over eating and consuming large amounts of food (and/or engaging in compensatory weight control behaviors) has on glycemic control and/or one’s perceived ability to effectively manage their eating/diabetes.

Past studies have found that diabetes distress is associated with eating disorder symptoms among individuals with diabetes [26, 29–31]. The current study adds to this preliminary data, and expands what is known by suggesting that diabetes distress may not only be a factor that increases risk, but is also a consequence that may maintain these behavior patterns. This may mean that explicitly targeting diabetes distress in eating disorder treatment, rather than narrowly focusing on body weight and shape concerns, could have benefits for this unique patient population.

Diabetes distress was a between-person factor significantly associated with OBE, but momentary elevations in diabetes distress did not explain additional variance in OBE episode risk. In our previous study, we found that diabetes distress tends to have less variability than other negative emotional states with participants’ diabetes distress levels remaining relatively constant when assessed multiple times a day [31]. This may indicate that individuals are less sensitive to subtle changes in diabetes distress and may benefit from treatments that increase capacity to observe fluctuations influencing momentary behavior. Diabetes distress may also be conflated with or influence other negative emotional states (or other negative emotional states may influence perceptions of diabetes distress). For example, participants may have been reporting anger when, perhaps with less awareness, underlying distress about diabetes was generating such anger (e.g., feeling angry that pre-meal blood sugar was out of range).

The results of the current study should be considered in light of its limitations. First, the 3-day assessment period may not have been a sufficient amount of time to capture patterns of emotional antecedents and consequences of binge eating. It may be that with additional time and more episodes of binge eating to analyze (either due to decreases in participant reactivity or simply more opportunities for binge eating to occur), different patterns may emerge. Second, we characterized OBE episodes based on participant report of whether or not an objectively large amount of food was consumed. While participants were trained on parameters defining an objectively large amount of food, patient perception may still have biased results by inaccurately classifying eating episodes. For example, participants who feel a lot of shame for eating may describe an episode as an objectively large amount of food despite it not meeting the provided definition. Third, the impact of OBE on postprandial blood glucose may have been underestimated in this study. We examined the effect of OBE on postprandial blood glucose irrespective of whether or not insulin restriction also occurred. Elevations in postprandial blood glucose may be even higher when insulin restriction follows OBE, which might commonly occur among some individuals. We also compared the effect of OBE episodes on postprandial blood glucose relative to all non-OBE eating episodes combined (i.e., non-OBE episodes included normal eating, overeating and subjective binge eating episodes). This may have further mitigated the relative effects of OBE on postprandial blood glucose by elevating postprandial blood glucose of non-OBE episodes. While an objectively large amount of food is by definition not consumed during subjective binge eating episodes, the experience of losing control over eating may still increase the risk of using insulin restriction for weight control resulting in subsequent postprandial blood glucose elevations [40]. In our previous study, feeling of loss of control over eating (regardless of whether an objectively large amount of food was consumed) was associated with insulin restriction (see Merwin et al., 2015 [31]). Lastly, the sample consisted mostly of White women with type 1 diabetes who reported elevated DEPS-R scores and is not a representative sample. Thus, we cannot say anything about frequency of OBE in the general population of type 1 diabetes patients or whether these patterns would generalize to other type 1 diabetes patients with OBE. This may limit the generalizability of the findings.

## Conclusions

Overall, findings indicate that individuals who tend to experience negative affect and diabetes distress before eating are at increased risk of binge eating at the upcoming meal. Engaging in binge eating may result in greater subsequent negative affect, including diabetes distress, and lead to elevated postprandial blood glucose levels. Although the current study cannot speak to causation, it is possible that the negative consequences of OBE may actually be a factor maintaining binge eating behavior. That is, individuals may engage in subsequent binge eating to cope with the emotional distress they experience following previous binge eating episodes (e.g., feeling distressed about elevations in blood glucose and/or their ability to effectively manage their eating/diabetes)*.* These findings add to a growing literature suggesting diabetes distress is related to eating disordered behaviors among individuals with type 1 diabetes and further suggest that it may have a role in maintaining the problem [21, 24–26]. Interventions that focus on helping individuals cope with negative affect and specifically diabetes distress may be helpful to incorporate into treatments for type 1 diabetes patients.

## Abbreviations

CGM: continuous glucose monitoring
DEPS-R: Diabetes Eating Problems Survey-Revised
OBE: objective binge eating
T1D: type 1 diabetes
